# Developing a weighted reward criterion for the Markov-based decision of road maintenance

**DOI:** 10.1186/s40064-016-2464-2

**Published:** 2016-06-16

**Authors:** Hui Gao, Xueqing Zhang, Yashuai Li

**Affiliations:** Department of Engineering Economics and Management, Institute of Construction Management, Hohai University, Nanjing, 210098 China; Department of Civil and Environmental Engineering, The Hong Kong University of Science and Technology, Hong Kong, China; School of Economics and Management, Beihang University, Beijing, 100191 China

**Keywords:** Maintenance, Decision analysis, Markov-based optimization, Weighted reward criterion, Life cycle costs

## Abstract

Reward criterion is an important decision factor in a Markov-based road maintenance optimization model. At present, average reward criterion or discounted reward criterion is widely used to optimize life cycle costs of road maintenance. However, the former one cannot reflect the time value of life cycle costs whereas the latter one tends to neglect the costs accumulated in the later periods over the decision horizon. In this regard, a weighted reward criterion is developed for the Markov-based road maintenance optimization model. It measures the trade-off of the average reward and the discounted reward by setting the weights of two rewards. In addition, the existence of the optimal plan under the weighted reward criterion is proven by two numerical examples under two scenarios with and without considering the inflation on maintenance costs. Finally, comparison is conducted between the proposed criterion and the average reward criterion/the discounted reward criterion to check the impacts of discount rates and inflation rates on the optimal plan.

## Background

To provide and distribute desired services of a road project, it is important to maintain road pavements in a good or at least serviceable performance over its service life. Unfortunately, due to road usage and environmental effects, the pavement performance of a road will gradually deteriorate with time. In this situation, different maintenance actions have come into effect to improve the pavement performance. A substantial amount of cost will unavoidably incur to take these measures. However, maintenance budgets of government agencies are always limited towards these maintenance actions. A road maintenance decision is thus a trade-off that balances the improvement of pavement performance and the expenditures of maintenance actions over the service life of a road project. As a result, this decision will be carried out on the basis of an accurate knowledge of modeling the change of pavement performance over the service life of a road project and an effective method of evaluating the rewards of maintenance actions on both the performance improvement and the costs (Hong and Hastak 2007; Yoon et al. 2014).

The pavement deterioration of a road segment can usually be modeled by a stochastic process, which holds the Markovian property. The Markovian property indicates that (1) the state of a road segment at a future time points only depends on its current state and the maintenance action to be taken; and (2) the future state of a road segment is independent of all its previous states and maintenance actions. In particular, the transition probability matrix describes the probabilities that a road segment will stay in the current state or transit to another state at the next time point when it holds a certain state and receives a maintenance action at the current time points. Based on the pavement deterioration modelling, the decision of road maintenance can be figured out in terms of the Markov decision process (MDP). The MDP is a stochastic control process that consists of the key aspects of decision epochs, states, actions, transition probabilities and rewards (Puterman 2005). Currently, some mathematical models have been proposed to manage roads and other infrastructure assets from the perspective of the MDP (Smilowita and Madanat 2000; Jiang et al. 2000; Ferreria et al. 2002; Guillaumot et al. 2003; Madanat et al. 2006; Zhang and Gao 2010, 2012a; Gao and Zhang 2013; Zhang et al. 2013; Adey et al. 2014).

In existing MDP models for road maintenance, the average reward criterion (Smilowita and Madanat 2000; Madanat et al. 2006) or the discounted reward criterion (Jiang et al. 2000; Guillaumot et al. 2003) is the commonly used reward criterion to find the optimal life cycle costs. On the one hand, average reward criterion tends to minimize the average costs over the service life and cannot capture the time value and the influence of the inflation on the maintenance decisions. On the other hand, discounted reward criterion considers the time value so that it emphasizes on the costs in the early periods and may neglect the costs accumulated in the later periods over the service life. In other words, average reward criterion is suitable for a long-term decision (e.g. more than 50 years) whereas discounted reward criterion is appropriate for a short-term decision (e.g. 3–5 years). However, the service life of a road project in general will be about 20–30 years, which is a time span between a short-term period and a long-term period. It is therefore necessary to tradeoff between the long-term average reward and the short-term discounted reward in the decision of road maintenance (Zhang and Gao 2012b).

In this paper, we first identify the pavement performance states and maintenance actions. Then, a weighted reward criterion, which considers both the average reward and the discounted reward, is developed for the Markov-based road maintenance optimization models over a finite decision horizon and an infinite decision horizon. The decisions are made at the network level to achieve a better result in terms of optimal utilization of resources and improved economies of scale than those made separately for individuals (Chi et al. 2013). In addition, two scenarios with and without considering the inflation over the decision horizon are taken into account in the illustrative example. The models using the two commonly used reward criteria and the proposed weighted reward criterion are further compared under two scenarios. The observations show that the effectiveness of the developed weighted reward criterion in the decision of road maintenance. Finally, the conclusions are given.

## Performance modeling of road pavement

### Performance indicator and performance states of road pavement

Roughness is a measure of pavement surface distortion that reflects the ability of the pavement to provide a comfortable ride to the users. Therefore, it is viewed as a primary consideration with respect to serviceability. Its structural deficiencies and accelerated pavement deterioration are largely due to vehicle operating costs, safety, comfort, and speed of travel. Roughness is traditionally measured by the International roughness index (IRI). However, IRI has unbounded value scopes such that there is no basis to classify the IRI into a certain number of performance states. In this paper, the IRI is converted into the Ride Quality Index (RQI) to measure the pavement performance for bituminous pavement (Gao and Zhang 2013). Based on the RQI, the performance of a road segment is indicated by five classified performance states. The set of possible states is expressed as *S* = {*s*_1_, *s*_2_, *s*_3_, *s*_4_, *s*_5_}, where *s*_1_ = excellent (4 ≤ RQI ≤ 5), *s*_2_ = good (3 ≤ RQI < 4), *s*_3_ = fair (2 ≤ RQI < 3), *s*_4_ = poor (1 ≤ RQI < 2), and *s*_5_ = unacceptable (0 ≤ RQI < 1). Table 1 presents the representative value of RQI and the corresponding IRI of each performance state for flexible pavement (Gao and Zhang 2013).

**Table 1:** Representative RQI and IRI values of each performance state (Gao and Zhang 2013)

Performance state	Range of RQI	Representative RQI value	Representative IRI value (m/km)
*s*_1_	4 ≤ RQI ≤ 5	4.5	0.683
*s*_2_	3 ≤ RQI < 4	3.5	1.784
*s*_3_	2 ≤ RQI < 3	2.5	3.405
*s*_4_	1 ≤ RQI < 2	1.5	5.544
*s*_5_	0 ≤ RQI < 1	0.5	8.202

### Effects of maintenance actions

According to the maintenance administrative handbook (Highways Department 2001), the major road maintenance actions usually are reconstruction, resurfacing, and routine maintenance (e.g., crack sealing and road cleansing). In this paper, without the loss of generality, all road maintenance actions are standardized as the aforementioned three types. That is to say, it is assumed that there are three alternative maintenance actions for any road segment in any state: *a*_1_ = reconstruction, *a*_2_ = resurfacing, *a*_3_ = do nothing. The action set *A* is expressed as*A* = {*a*_1_, *a*_2_, *a*_3_}. Different actions have different effects: “Reconstruction” can improve a road segment to the excellent state; “do nothing” is considered to have no effect on the road performance; and the effect of a resurfacing work can be estimated by the reduction of IRI in Gao and Zhang (2013).

## Markov-based road maintenance decision model using weighted reward criterion

### Decision epochs and decision periods

Decision epochs are the time points at which the decisions are made. In the MDP, the decision horizon is divided into *M* periods by decision epochs. If *M* is infinite, the decision is made on an infinite horizon. Otherwise, the decision is made on a finite horizon if *M* is finite. It is generally assumed that decisions are made annually. That is, the decision period is 1 year and the decision epoch is the beginning of each year. Also, we assume that all maintenance actions are conducted at the beginning of each year.

### Weighted reward criterion

A weighted reward criterion consists of a weighted combination of the average reward criterion and the discounted reward criterion. The decision maker can pay more or less emphasis on the long-term reward versus short-term reward by changing their associated weights. Krass et al. (1992) presented a general formula as shown in Eq. (1) to calculate the weighted reward in terms of the average reward and the discounted reward. This “weighted reward” is a convex combination of the average reward and discounted reward by varying their weights (Krass et al. 1992).

1 $$C_{W} = \alpha C_{E} + \beta (1 - \lambda )C_{D}$$

where *C*_*W*_ = weighted reward; *C*_*E*_ = average reward; *C*_*D*_ = discounted reward; *α* = weight of average reward; *β* = weight of discounted reward, *α* + *β* = 1; and *λ* = (1 + *r*)^−1^, *λ* < 1, *r* = discount rate.

### Optimization models based on the weighted reward criterion

In this section, an optimization model using the developed weighted reward criterion is first formulated to minimize the expected life cycle maintenance costs over a finite decision horizon. The decision variables are the distribution of road segments associated with each state-action pair [a state-action pair (*i*, *a*) means that a maintenance action *a* is taken when the segment is in state *i*] at the beginning of each year over a finite decision horizon. Then, an infinite-time model will be developed to extend the optimization to an infinite decision horizon. The results show that the model using the developed weighted reward criterion will converge to the model using the average reward criterion if the decision horizon tends to be infinite.

#### Finite-time optimization model

The finite-time model seeks an optimal distribution of road segments for each state-action pair that minimizes the expected life cycle road maintenance costs over a finite decision horizon. The objective functions of finite-time MDP models using the criteria of average reward and discounted reward are formulated as follows:

2 $${\text{Min }}C_{E} (\pi )_{\text{finite}} = \frac{1}{T}\sum\limits_{t = 1}^{T} {\sum\limits_{i \in S} {\sum\limits_{a \in A} {Nc_{t} (i,a)d_{t} (i,a)} } }$$

3 $${\text{Min }}C_{D} (\pi )_{\text{finite}} = \sum\limits_{t = 1}^{T} {\sum\limits_{i \in S} {\sum\limits_{a \in A} {Nc_{t} (i,a)\lambda^{t - 1} d_{t} (i,a)} } }$$

where *C*_*E*_(*π*) = the expected average life cycle costs of maintenance plan *π*; *C*_*D*_(*π*) = the expected discounted life cycle costs of maintenance plan *π*; *c*_*t*_(*i*, *a*) = maintenance cost associated with state-action pair (*i*, *a*) on road segments in year *t*; *d*_*t*_(*i*, *a*) = distribution of road segments in state-action pair (*i*, *a*) at the beginning of year *t*; *T* = decision horizon; *N* = number of road segments; *S* = state space; and *A* = action set.

According to Eq. (1), the objective function of a finite-time road maintenance optimization model using the weighted reward criterion is formulated as:

4 $$\begin{aligned} {\text{Min }}C_{W} (\pi )_{\text{finite}} &= \alpha C_{E} (\pi )_{\text{finite}} + \beta (1 - \lambda )C_{D} (\pi )_{\text{finite}} \\ &=\sum\limits_{t = 1}^{T} {\sum\limits_{i \in S} {\sum\limits_{a \in A} {N\left[ {\alpha \frac{1}{T}c_{t} (i,a)d_{t} (i,a) + \beta (1 - \lambda )c_{t} (i,a)\lambda^{t - 1} d_{t} (i,a)} \right]} } } \end{aligned}$$

where *C*_*W*_(*π*) = the expected life cycle costs of maintenance plan *π* using the weighted reward criterion.

The decision variables of a finite-time model are the road segment distributions in each state-action pair at the beginning of each year, which is dependent of the initial state distribution. The model constraints on the road segment distribution, state transition, available budget and performance requirement are described as follows:

1. The distribution of road segments in each state-action pair should be non-negative:
  5 $$d_{t} (i,a) \ge 0 \quad \forall \, i \in S, \, a \in A, \quad t = 1, \, 2, \ldots , \, T$$
2. The initial road segment distribution of state *i* is specified as:
  6 $$\sum\limits_{a \in A} {d_{1} (i,a)} = d_{1} (i) \quad \forall \, i \in S$$
  where *d*_1_(*i*) = initial road segment distribution of state *i*;
3. The summation of road segment distributions in all state-action pairs at the beginning of year *t* should be equal to 1:
  7 $$\sum\limits_{i \in S} {\sum\limits_{a \in A} {d_{t} (i,a)} } = 1 \quad \forall \, t = 1, \, 2, \ldots , \, T$$
4. The state transition should satisfy the following equation:
  8 $$\sum\limits_{a \in A} {d_{t} (i,a)} = \sum\limits_{j \in S} {\sum\limits_{a \in A} {d_{t - 1} (j,a)p_{ji} (a)} } \quad \forall \, i \in S, \, t = 2, \ldots , \, T$$
  where *p*_*ji*_(*a*) = the transition probability of a road segment from state *j* to state *i* when maintenance action *a* is taken;
5. Budget constraints (the average annual maintenance budget for the road):
  9 $$\sum\limits_{i \in S} {\sum\limits_{a \in A} {Nc_{t} (i,a)d_{t} (i,a)} } \le B_{t} \quad \forall \, t = 1, \, 2, \ldots , \, T$$
  where *B*_*t*_ = available budget of year *t*;
6. Performance requirements (the minimum RQI to be maintained for the road):
  10 $$\sum\limits_{i \in S} {\sum\limits_{a \in A} {r(i)d_{t} (i,a)} } \ge R_{t} \quad \forall \, t = 1, \, 2, \ldots , \, T + 1$$
  where *R*_*t*_ = the minimum RQI to be maintained in year *t*; and *r*(*i*) = the representative RQI of state *i*.

#### Infinite-time optimization model

In an infinite-time maintenance optimization model, the annual maintenance cost is constant over the service life of a road project. Given that the number of road segments is *N* and the decision horizon tends to be infinite, according to Eqs. (2) and (3), the objective functions of the infinite-time models using the average reward criterion and the discounted reward criterion can be written as follows:

11 $$\begin{aligned} {\text{Min }}C_{E} (\pi )_{\text{infinite}} & = \mathop {\lim }\limits_{T \to \infty } \frac{1}{T}\sum\limits_{t = 1}^{T} {\sum\limits_{i \in S} {\sum\limits_{a \in A} {Nc(i,a)d(i,a)} } } \\ & = \sum\limits_{i \in S} {\sum\limits_{a \in A} {Nc(i,a)d(i,a)} }\end{aligned}$$

12 $$\begin{aligned} {\text{Min }}C_{D} (\pi )_{\text{infinite}} & = \mathop {\lim }\limits_{T \to \infty } \sum\limits_{t = 1}^{T} {\sum\limits_{i \in S} {\sum\limits_{a \in A} {Nc(i,a)\lambda^{t - 1} d(i,a)} } } \\ & = \frac{1}{1 - \lambda }\sum\limits_{i \in S} {\sum\limits_{a \in A} {Nc(i,a)d(i,a)} } \end{aligned}$$

where *c*(*i*, *a*) = annual maintenance cost associated with state-action pair (*i*, *a*) on segments; and *d*(*i*, *a*) = annual distribution of road segments that are in state-action pair (*i*, *a*).

According to Eq. (1), the objective function of the infinite-time model using the weighted reward criterion is established as follows:

13 $$\begin{aligned} {\text{Min }}C_{W} (\pi )_{\text{infinite}} &= \alpha C_{E} (\pi )_{\text{infinite}} + \beta (1 - \lambda )C_{D} (\pi )_{\text{infinite}} \\ & = \sum\limits_{i \in S} {\sum\limits_{a \in A} {N\left[ {\alpha c(i,a)d(i,a) + \beta (1 - \lambda )c(i,a)\frac{d(i,a)}{(1 - \lambda )}} \right]} } \\ &= \sum\limits_{i \in S} {\sum\limits_{a \in A} {Nc(i,a)d(i,a)} } \hfill \\ \end{aligned}$$

It is found that Eq. (13) is equal to the objective function of the infinite-time model using the average reward criterion as shown in Eq. (11). That is to say, the model using the weighted reward is equal to the model using the average reward when the decision horizon tends to be infinite. This result proves the developed weighted reward criterion for the finite-time model is feasible because the average reward criterion is the most appropriate for the infinite decision horizon.

### Optimal maintenance plan

The optimal maintenance plan over the decision horizon can be denoted as a sum of *π*_*t*_(*i*, *a*) at the beginning of each year. It is calculated as follows:

14 $$\pi_{t} (i,a) = \frac{{d_{t} (i,a)}}{{\sum\limits_{a \in A} {d_{t} (i,a)} }} \quad \forall \, i,a,t$$

where *π*_*t*_(*i*, *a*) = the distribution of road segments in state *i* for which maintenance action *a* is taken at the beginning of year *t*.

## Illustrative example

In this paper, the maintenance of Lung Cheung Road, which is a part of Route 7 Expressway in Hong Kong, will be used as an example to illustrate the proposed decision model.

### Model inputs

#### Initial state distributions of road segments

The total length of the selected road section is 10 km. The road has dual three-lane with 4 m wide for each lane. Each road segment occupies three lanes and the length of each segment is 50 m. The area of each segment is 600 m^2^. The number of road segments is 400. Road pavement is asphalt concrete. All road segments are assumed to have similar deterioration processes. The representative RQI and IRI values as shown in Table 1 are used to calculate the average performance of road segments. The initial state distributions of road segments are listed in Table 2. The initial RQI of the road section is 4. The annual minimum performance requirement of the road section on RQI value is 3.5, which is assumed to be constant over the decision horizon. The transition probabilities are referred to Gao and Zhang (2013) and shown in Table 3.

**Table 2:** Initial road segment distributions in each state

State	*s*_1_	*s*_2_	*s*_3_	*s*_4_	*s*_5_	Total
Distribution of road segments	60 %	30 %	10 %	0	0	100 %
Number of road segments	240	120	40	0	0	400

**Table 3:** Transition probabilities of alternative maintenance actions

	*a*_1_					*a*_2_					*a*_3_				
	*s*_1_	*s*_2_	*s*_3_	*s*_4_	*s*_5_	*s*_1_	*s*_2_	*s*_3_	*s*_4_	*s*_5_	*s*_1_	*s*_2_	*s*_3_	*s*_4_	*s*_5_
*s*_1_	0.74	0.26	0	0	0	0.74	0.26	0	0	0	0.74	0.26	0	0	0
*s*_2_	0.74	0.26	0	0	0	0.74	0.26	0	0	0	0	0.82	0.18	0	0
*s*_3_	0.74	0.26	0	0	0	0.28	0.61	0.11	0	0	0	0	0.86	0.14	0
*s*_4_	0.74	0.26	0	0	0	0	0.19	0.7	0.11	0	0	0	0	0.87	0.13
*s*_5_	0.74	0.26	0	0	0	0	0	0.09	0.8	0.11	0	0	0	0	1

#### Costs of alternative maintenance actions

A “reconstruction” action involves the reconstruction of a subgrade layer, a sub-base layer, a 200 mm base course of crushed rock, and a 60 mm asphalt layer. A “resurfacing” action involves the placement of a 40 mm asphalt overlay. The estimated costs for “reconstruction” and “resurfacing” are HK $390/m^2^ and HK $150/m^2^, respectively. The “do nothing” is assumed to be no expense. Table 4 shows the maintenance costs of alternative maintenance actions for individual road segment.

**Table 4:** Maintenance costs of alternative maintenance actions for individual road segment

Maintenance action	*a*_1_	*a*_2_	*a*_3_
Maintenance cost (HK $)	234,000	90,000	0

### Analysis scenarios

In this paper, we analyze two scenarios with and without considering the inflation over the decision horizon, which is set on 30 years.

- *Scenario 1* The costs and budgets are assumed to be constant over the decision horizon. The costs of maintenance actions “*a*_1_”, “*a*_2_” and “*a*_3_” are shown in Table 4.
- *Scenario 2* The costs and budgets are assumed to be annually increased with an inflation rate. Similarly, the annual budget available for the selected road section will be annually increased with the same inflation rate. The base costs and budget of first year are equal to the constant values in Scenario 1.

### Model outputs

#### Optimal annual maintenance budget

**Fig. 1:**
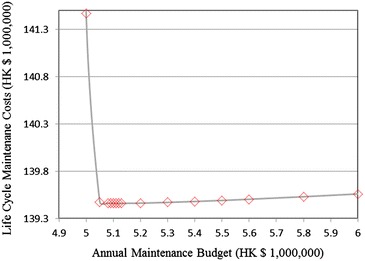
Expected life cycle cost with the change of annual maintenance budget

In this example, we first test the maintenance budget with a gradient HK $ 10,000 to find the optimal annual budget with the assumption that the costs and budgets are constant over the decision horizon. According to the test results, the minimum required annual budget is HK $ 4,990,000. If the budget is lower than this value, the model cannot obtain a feasible solution. When the budget is increased from the minimum required budget, the expected life cycle cost will be steeply decreased to touch the bottom. Then, it will be mildly increased if the budget keeps increasing. The results are shown in Fig. 1. The optimal annual maintenance budget is HK $5,120,000.

#### Outputs of scenario 1

In scenario 1, decision models using three reward criteria are solved and compared when the maintenance budget is HK $5,120,000. Following results are observed:

- When the average reward criterion is used, the annual state distributions tend to be steady state distributions around the beginning of the 20th year, which is almost same to the optimal state distributions obtained from the infinite-time model as shown in Table 5. That is to say, in this example, the model with a decision horizon over 20 years tends to be an infinite-time model. In this case, the decision using the weighted reward is equal to the decision using the average reward which is explained by Eq. (13).

**Table 5:** Steady state distributions obtained from the infinite-time model

	*s*_1_	*s*_2_	*s*_3_	*s*_4_	*s*_5_
*a*_1_	0	0	0	0	0
*a*_2_	0	0	0.139	0	0
*a*_3_	0.149	0.709	0	0.003	0
- When the discounted reward criterion is used and the discount rate is set on 5 %, the minimized life cycle costs are same to those obtained from the model using the average reward criterion. That is to say, the optimal state distributions of road pavements at the beginning of each year obtained from two models are same. When the discount rate is a larger one, e.g. 20 %. The difference of minimized life cycle costs in two models are HK $25 (HK $139,460,364 and HK $139,460,389 for the average reward and the discounted reward), which is very tiny. The optimal state distributions are almost same. The impact of the discount rate on the optimal state distributions can be neglected if the model tends to be an infinite-time model.
- From the above observations, it can be concluded that the optimal state distributions obtained from decision models using three reward criteria are same if a finite-time model tends to be an infinite-time model. That is to say, the average reward is preferred in the maintenance decision. Table 6 shows the optimal state distributions of road segments at the beginning of each year (only the year 5, 10, 15, 20, 25 and 30 are shown to save the space) obtained from the model using the weighted reward criterion.

**Table 6:** Optimal state distributions under the weighted reward criterion in scenario 1

	*s*_1_	*s*_2_	*s*_3_	*s*_4_	*s*_5_	*s*_1_	*s*_2_	*s*_3_	*s*_4_	*s*_5_	*s*_1_	*s*_2_	*s*_3_	*s*_4_	*s*_5_
	5th year					10th year					15th year				
*a*_1_	0	0	0	0	0.005	0	0	0	0	0.002	0	0	0	0	0.001
*a*_2_	0	0	0.128	0	0	0	0	0.134	0.001	0	0	0	0.137	0	0
*a*_3_	0.240	0.573	0.011	0.043	0	0.168	0.682	0	0.013	0	0.153	0.702	0.001	0.006	0

	20th year					25th year					30th year				
*a*_1_	0	0	0	0	0.001	0	0	0	0	0	0	0	0	0	0
*a*_2_	0	0	0.138	0	0	0	0	0.139	0	0	0	0	0.139	0	0
*a*_3_	0.150	0.705	0.002	0.004	0	0.149	0.707	0.002	0.003	0	0.149	0.707	0.002	0.003	0

#### Outputs of scenario 2

In Scenario 2, the inflation is involved and tested from 1 to 10 %. Decision models using three reward criteria are solved and compared when the base maintenance budget at the beginning of first year is HK $5,120,000. Following results are further observed:

- The minimized life cycle costs of the model using the average reward criterion and the model using the discounted reward criterion (two discount rates, i.e., 5 and 10 % are used) are same if the inflation rate is lower than or equal to 3 %. When the inflation rate is larger than 3 %, the outputs are different. The results are shown in Table 7. It means that, in this case, the optimal state distributions obtained from decision models using three different reward criteria will not be same when the inflation rate on the costs and budget is larger than 3 %.

**Table 7:** Minimized life cycle costs under various discount rates and inflation rates

Discount rate (%)	Inflation rate							
	1 %	2 %	3 %	4 %	5 %	7 %	8 %	10 %
0	163,432,436	192,419,226	227,555,101	270,225,976	322,045,264	461,706,709	555,546,583	810,921,234
5	163,432,436	192,419,226	227,555,115	270,236,573	322,182,333	462,811,648	557,292,986	813,218,613
10	163,432,436	192,419,226	227,555,115	270,236,573	322,182,333	462,811,648	557,293,138	814,393,543
- When the inflation rate is lower than or equal to 7 %, the minimized life cycle costs obtained from two models using two different discount rates are same. When the inflation rate is larger than 7 %, the outputs are different. The results are also shown in Table 7. It shows that the discount rate has a larger impact on the optimal state distributions due to the existence of the inflation on the costs and budget.
- In terms of those two observations, the paper solves the model using the weighted reward criterion with assuming that the weights of the average reward and the discounted reward in the optimization model are 0.7 and 0.3. The discount rate is 10 %. The inflation rate is 5 %. Table 8 shows the optimal state distributions of road segments at the beginning of each year (only the year 5, 10, 15, 20, 25 and 30 are shown to save the space).

**Table 8:** Optimal state distributions under the weighted reward criterion in scenario 2

	*s*_1_	*s*_2_	*s*_3_	*s*_4_	*s*_5_	*s*_1_	*s*_2_	*s*_3_	*s*_4_	*s*_5_	*s*_1_	*s*_2_	*s*_3_	*s*_4_	*s*_5_
	5th year					10th year					15th year				
*a*_1_	0	0	0	0	0.004	0	0	0	0	0.001	0	0	0	0	0.001
*a*_2_	0	0	0.133	0	0	0	0	0.139	0	0	0	0	0.139	0	0
*a*_3_	0.237	0.582	0.007	0.037	0	0.148	0.69	0.003	0.019	0	0.139	0.709	0.004	0.008	0

	20th year					25th year					30th year				
*a*_1_	0	0	0	0	0	0	0	0	0	0	0	0	0	0	0
*a*_2_	0	0	0.141	0	0	0	0	0.142	0	0	0	0	0.143	0	0
*a*_3_	0.136	0.715	0.003	0.005	0	0.133	0.72	0.001	0.004	0	0.132	0.722	0.001	0.002	0
- The minimized life cycle costs of models using the average reward criterion and the discounted reward criterion are HK $ 322,045,264 and 322,182,333, respectively. The minimized life cycle cost of models using the weighted reward criterion is HK $ 321,952,277. It shows that the weighted reward criterion is more suitable than the other two criteria.

### Optimal maintenance plan

Based on the state distribution as shown in Table 8 and Eq. (14), the optimal policy for the road segments in each year of the 30-year planning horizon can be obtained, which is shown in Table 9. It is noted that *π*_*t*_(*i*, *a*) in Eq. (14) specify a distribution of road segments in state *i* on which maintenance action *a* will be taken. That is to say, a road segment that are in state *i* may have one or more selection of the maintenance actions. However, this randomness is limited. In the most cases, the selection of maintenance action for a road segments in a state is limited to one. In Table 9, the value “1” for state-action pair (*s*_1_, *a*_3_) means that the probability to take the action *a*_3_ is 1 when a segment stays in the state *s*_1_. It is observed that the randomness of selecting “resurfacing” and “do nothing” is only existed in state *s*_3_, in which the “resurfacing” is the major choice. In other states, there is only one choice in selecting maintenance actions. Thus, the optimal maintenance plan is feasible in the actual maintenance decision.

**Table 9:** Optimal road maintenance plan

	*s*_1_	*s*_2_	*s*_3_	*s*_4_	*s*_5_	*s*_1_	*s*_2_	*s*_3_	*s*_4_	*s*_5_	*s*_1_	*s*_2_	*s*_3_	*s*_4_	*s*_5_
	5th year					10th year					15th year				
*a*_1_	0	0	0	0	1	0	0	0	0	1	0	0	0	0	1
*a*_2_	0	0	0.95	0	0	0	0	0.979	0	0	0	0	0.972	0	0
*a*_3_	1	1	0.05	1	0	1	1	0.021	1	0	1	1	0.028	1	0

	20th year					25th year					30th year				
*a*_1_	0	0	0	0	1	0	0	0	0	1	0	0	0	0	1
*a*_2_	0	0	0.979	0	0	0	0	0.993	0	0	0	0	0.993	0	0
*a*_3_	1	1	0.021	1	0	1	1	0.007	1	0	1	1	0.007	1	0

## Conclusions

Markov-based optimization models using the average reward criterion or the discounted reward criterion are widely utilized in current road maintenance. However, both of the two reward criteria have deficiencies in modeling a road project whose service life commonly is 20–30 years. In this regard, a weighted reward criterion is developed to balance both the average reward and the discounted reward. The illustrative example analyzes two scenarios with and without considering the inflation over the decision horizon. When the inflation is not considered and in case of a finite-time model tends to be an infinite-time model, the average reward is preferred and the optimal state distributions obtained from decision models using three reward criteria are same. However, if the inflation is considered, a finite-time model cannot tend to be an infinite-time model due to the inflation rate. The optimal state distributions obtained from decision models using three reward criteria are different from each other. In particular, the model using the weighted reward criterion could get the smallest life cycle cost. It means that the weighted reward criterion is more suitable than the other two commonly used reward criteria. In addition, the example also proves the existence of optimal road maintenance plan under the weighted reward criterion.
